# Endophytic Bacterium *Pseudomonas fluorescens* RG11 May Transform Tryptophan to Melatonin and Promote Endogenous Melatonin Levels in the Roots of Four Grape Cultivars

**DOI:** 10.3389/fpls.2016.02068

**Published:** 2017-01-10

**Authors:** Yaner Ma, Jian Jiao, Xiucai Fan, Haisheng Sun, Ying Zhang, Jianfu Jiang, Chonghuai Liu

**Affiliations:** ^1^Zhengzhou Fruit Research Institute, Chinese Academy of Agricultural SciencesZhengzhou, China; ^2^College of Enology, Northwest A&F UniversityYangling, China

**Keywords:** melatonin, plant growth-promoting bacteria, root crude extracts, salt stress, grapevine

## Abstract

Endophytes have been verified to synthesize melatonin *in vitro* and promote abiotic stress-induced production of endogenous melatonin in grape (*Vitis vinifera* L.) roots. This study aimed to further characterize the biotransformation of tryptophan to melatonin in the endophytic bacterium *Pseudomonas fluorescens* RG11 and to investigate its capacity for enhancing endogenous melatonin levels in the roots of different grape cultivars. Using ultra performance liquid chromatography-tandem mass spectrometry combined with 15N double-labeled *L*-tryptophan as the precursor for melatonin, we detected isotope-labeled 5-hydroxytryptophan, serotonin, *N*-acetylserotonin, and melatonin, but tryptamine was not detected during the *in vitro* incubation of *P. fluorescens* RG11. Furthermore, the production capacity of these four compounds peaked during the exponential growth phase. RG11 colonization increased the endogenous levels of 5-hydroxytryptophan, *N*-acetylserotonin, and melatonin, but reduced those of tryptamine and serotonin, in the roots of the Red Globe grape cultivar under salt stress conditions. Quantitative real-time PCR revealed that RG11 reduced the transcription of grapevine tryptophan decarboxylase and serotonin *N*-acetyltransferase genes when compared to the un-inoculated control. These results correlated with decreased reactive oxygen species bursts and cell damage, which were alleviated by RG11 colonization under salt stress conditions. Additionally, RG11 promoted plant growth and enhanced the levels of endogenous melatonin in different grape cultivars. Intraspecific variation in the levels of melatonin precursors was found among four grape cultivars, and the associated root crude extracts appeared to significantly induce RG11 melatonin biosynthesis *in vitro*. Overall, this study provides useful information that enhances the existing knowledge of a potential melatonin synthesis pathway in rhizobacteria, and it reveals plant–rhizobacterium interactions that affect melatonin biosynthesis in plants subjected to abiotic stress conditions.

## Introduction

During their evolution, most land plants, developed symbiotic associations with microbes, including fungi, bacteria, and actinomycetes that can grow in the roots and other plant tissues (Compant et al., 2011; Hameed et al., 2015; Wang et al., 2016). Numerous plant-associated microbes are beneficial components of plant micro-ecosystems (Chebotar et al., 2015) because they provide protection against phytopathogens (Lindow and Brandl, 2003; Jetiyanon, 2007), enhance mineral nutrient acquisition (Elbeltagy et al., 2001; Johnson, 2008; Richardson et al., 2009), and help plants withstand abiotic stresses (Castiglioni et al., 2008; Zhang et al., 2008; Khan et al., 2015). The most researched role of plant-associated microbes is their potential to regulate plant growth by acting as suppliers of diverse phytohormones, including gibberellins (Bottini et al., 2004; Shahzad et al., 2016), indole acetic acid (Patten and Glick, 1996; Etesami et al., 2014), cytokinins (Ie et al., 2001; Arkhipova et al., 2007), jasmonates and abscisic acid (Forchetti et al., 2007; Belimov et al., 2014; Salomon et al., 2014).

We previously reported that several common endophytic bacteria have the capacity to synthesize the additional plant physiology regulator, melatonin (*N*-acetyl-5-methoxytryptamine), and could promote the abiotic stress-induced production of endogenous melatonin in grape roots (*Vitis vinifera* L.) (Jiao et al., 2016). This indoleamine molecule was first isolated from the bovine pineal gland (Lerner et al., 1958), and is now recognized as a ubiquitous compound among living organisms including humans, animals, plants, bacteria, fungi, and macroalgae (Tilden et al., 1997; Rodriguez-Naranjo et al., 2012; Tan et al., 2012). Melatonin was first identified in edible plants in 1995 (Dubbels et al., 1995; Hattori et al., 1995), and it was subsequently identified in hundreds of plant species (Burkhardt et al., 2001; Reiter et al., 2005; Okazaki and Ezura, 2009; Murch et al., 2010). Exogenous melatonin can act as a phytoregulator of seed germination (Zhang et al., 2013, 2014), flowering (Kolář et al., 2003), fruit ripening, anthocyanin accumulation (Sun et al., 2016), root system architecture (Pelagio-Flores et al., 2012), chlorophyll preservation, and leaf senescence (Zhang et al., 2016). It is also a powerful antioxidant that directly decreases the levels of ROS or indirectly modulates antioxidant enzyme activities (Posmyk et al., 2008, 2009; Arnao and Hernández-Ruiz, 2009; Nawaz et al., 2016). Abiotic stressors can elevate the levels of endogenous melatonin in plants (Arnao and Hernández-Ruiz, 2013a,b, 2016; Shi et al., 2015), so stress-induced ROS may trigger melatonin accumulation (Arnao and Hernández-Ruiz, 2015).

Because melatonin has multiple functions, more information regarding the melatonin-producing mechanism in endophytic bacteria and bacterial interactions with plants via melatonin is needed. Unlike exogenous melatonin application, melatonin-producing endophytes might have long-term effects on endogenous melatonin levels in plants once they enter plant tissues. In vertebrates, the biosynthesis of tryptophan to melatonin is well described, and its synthetic pathway involves three main intermediates, 5-hydroxytryptophan, serotonin, and *N*-acetylserotonin, which are catalyzed by tryptophan hydroxylase, aromatic amino acid decarboxylase, and animal serotonin *N*-acetyltransferase, respectively (Tan et al., 2015). However, this pathway in plants differs markedly from that in vertebrates (Tan et al., 2014) in that plants initially decarboxylate tryptophan to form tryptamine with tryptophan decarboxylase, and hydroxylate tryptamine is subsequently used to form serotonin with tryptamine 5-hydroxylase (Byeon and Back, 2013; Byeon et al., 2014b). It is speculated that the melatonin synthetic machinery in eukaryotes was inherited from bacteria as a result of endosymbiosis (Tan et al., 2013); however, the melatonin synthetic mechanism in bacteria remains unclear. Additionally, we observed that the levels of endogenous melatonin in grape roots were enhanced by the colonization of melatonin-producing endophytic bacteria under abiotic stress conditions (Jiao et al., 2016), but the internal and external elements that could influence melatonin production need further study.

Among the abiotic stresses, salt stress is a major problem for agricultural lands located near coastal regions. In addition to the use of traditional breeding and plant genetic engineering, the use of melatonin-producing endophytes might be useful for the development of strategies that facilitate plant growth under salt stress conditions (Mayak et al., 2004). Here, we used the melatonin-producing endophytic bacterium *Pseudomonas fluorescens* RG11 to characterize the bacterial melatonin synthetic pathway using 15N double-labeled *L*-tryptophan as the melatonin precursor. The roots of four grape cultivars were inoculated with RG11 in order to: (i) investigate changes in ROS accumulation levels, endogenous melatonin levels, and related gene expression; and (ii) detect differences in root colonization, growth promotion, and endogenous melatonin levels among cultivars subjected to salt stress conditions. Finally, we compared the levels of melatonin intermediates in the roots of grape cultivars and further determined whether the root crude extracts could influence melatonin biosynthesis in RG11 *in vitro*.

## Materials and Methods

### Microorganism and Culture Conditions

**FIGURE 1:**
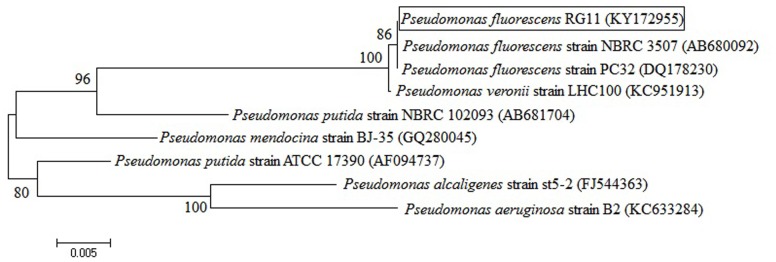
**Neighbor-joining dendrogram based on 16S rRNA gene sequences that were available in the GenBank database**. The tree was constructed using the MEGA 6.06 package. Bootstrap values were based on 1,000 replications and are listed as percentages at the nodes. The scale bar indicates genetic distance.

The *P. fluorescens* RG11 strain, which was previously isolated from the roots of the *V. vinifera* Red Globe grape cultivar, was used in this study. The root surfaces were sterilized to ensure that bacteria were isolated from internal tissues. A preliminary test showed that RG11 was able to produce melatonin *in vitro* and *P. fluorescens* was identified using morphological and biochemical characteristics according to Bergey’s Manual of Determinative Bacteriology (Krieg and Holt, 1984) and using a 16S rRNA sequencing analysis. The *P. fluorescens* 16S rDNA gene sequence (GenBank accession no. KY172955) was amplified using the universal bacterial primer pairs 27F/1492R in accordance with previously described conditions (Bai et al., 2002), and a neighbor-joining dendrogram generated by the program MEGA 6.06 indicated close similarity to *P. fluorescens* (**Figure 1**).

The RG11 strain was maintained at 4°C on nutrient agar slants (3 g L^-1^ beef extract, 10 g L^-1^ tryptone, 5 g L^-1^ NaCl, and 20 g L^-1^ agar; pH 7.4). To prepare inoculum cultures, cells from one colony were transferred to 50 mL nutrient broth medium and cultivated for 12–16 h at 30°C with an agitation of 150 rpm to a final OD_600_ of approximately 1.0. The cultures were then centrifuged at 6,000 × *g* for 10 min, re-suspended, and standardized to 1 × 10^8^ cells mL^-1^ in 0.9% sterilized saline solution using a Petroff–Hausser counting chamber.

### Measurement of the Ability of RG11 to Secrete Melatonin and Its Intermediates Using 15N Double-Labeled *L*-Tryptophan

To characterize melatonin biosynthesis in RG11, 1 mL of standardized bacterial inoculum was added to individual 100-mL brown bottles that contained 50 mL of nutrient broth (3 g L^-1^ beef extract, 10 g L^-1^ tryptone, and 5 g L^-1^ NaCl; pH 7.4) with 200 mg L^-1^ 15N double-labeled *L*-tryptophan. The cultures were incubated in a rotary shaker at 28°C with a rotational speed of 150 rpm in the dark under aerobic conditions. Samples were collected every 6 h for viable cell counts using the plate counting method and appropriate dilutions. A 1-mL aliquot of the bacterial culture was centrifuged, and the supernatant was diluted 1:1 with methanol. The resulting mixture was passed through a 0.22-μm filter, and was then used for the detection and quantification of ^15^N-tryptamine, ^15^N-5-hydroxytryptophan, ^15^N-serotonin, ^15^N-*N*-acetylserotonin, and ^15^N-melatonin using UPLC-MS/MS. The concentrations of these compounds were determined using an eight-point calibration curve of non-isotopic labeling standards as described by Jiao et al. (2016). The experiment was repeated in triplicate.

### Greenhouse Experiment and Microbial Inoculation

*Vitis vinifera* ‘Red Globe grape’ plantlets, obtained from an *in vitro* culture system, were grown in sterilized 500-mL glass bottles filled with 1/2 MS modified basal salt mixture (PhytoTechnology Laboratories, Shawnee Mission, KS, USA), which contained 3% sucrose, 0.6% agar, and 0.2 mg L^-1^ indolebutyric acid. Plantlets were grown at 28°C with a with a 16-h light/8-h dark cycle for 6 weeks. Plantlets of approximately 8 cm in height and with six leaves were selected and standardized to four 5-cm adventitious roots using sterilized scissors. The roots of some plantlets were immersed in the RG11 bacterial inoculum for 1 min (E+), whereas others were immersed in 0.9% sterile saline solution (E-). All plantlets were transferred to 500-mL culture bottles that contained 150 g sterile nutrient soil (Pindstrup, Ryomgaard, Denmark) and 40 mL nutrient-rich water that was prepared from 1/2 MS. The bottles were randomly placed in a controlled chamber at 26°C with 70% humidity under a 16-h light/8-h dark cycle, and plantlets were irrigated with 5 mL distilled water every 2 days.

After 20 days, 12 plantlets from each group (E+ and E-) were randomly selected for root length, root fresh weight (FW), lateral root number, plant height, and chlorophyll content measurements. The chlorophyll content of fully expanded leaves was analyzed using a chlorophyll ELISA kit, according to the manufacturer’s instructions (Lvyuan, Beijing, China). Both E+ and E- groups were divided into two subgroups (at least 84 plantlets each), and were watered either normally or with 20 mL of 80 mM NaCl solution. Roots were collected from 12 randomly selected plantlets from each subgroup between 9:00 AM and 10:00 AM daily. The roots were ground into powder in liquid nitrogen in individual mortars, and 0.5 g samples were extracted with 2 mL methanol as previously described (Boccalandro et al., 2011). The extracts were mixed with 2 mL ultrapure water, centrifuged, passed through a 0.22-μm filter, and stored in amber vials for the detection and quantification of 5-hydroxytryptophan, tryptamine, serotonin, *N*-acetylserotonin, and melatonin with UPLC-MS/MS. Other samples were used for the determination of MDA content and H_2_O_2_ accumulation.

To investigate whether the grape genotype could influence bacterial colonization and melatonin production, RG11 was used to inoculate the roots of three additional grape cultivars, including Riesling, Chardonnay, and Cabernet Sauvignon. We used the procedure described above with the following two modifications: (i) after measuring the growth attributes of each plantlet, the whole root was collected to assess bacterial colonization ability; and (ii) after exposure to 80 mM NaCl stress for 4 days, the plantlet roots were sampled between 9:00 AM and 10:00 AM for subsequent UPLC-MS/MS analyses. To assess bacterial colonization ability, roots were surface-sterilized with 70% ethanol for 3 min, soaked in sodium hypochlorite (3% available chlorine) for 2 min, and rinsed three times with sterile water. Then, 0.5 g of the root samples were ground and homogenized in 1.5 mL ice-cold phosphate-buffer saline solution using sterile quartz sand in individual mortars. The homogenates were serially diluted, spread, and incubated on Luria–Bertani agar at 30°C for 2–3 days to determine viable bacterial counts.

### Measurement of RG11 Melatonin Production *In vitro* with Crude Root Extracts

After exposure to 80 mM NaCl stress for 4 days, 10 g of root samples from E- Red Globe, Riesling, Chardonnay, and Cabernet Sauvignon plantlets were collected, washed three times with sterile H_2_O, ground, and homogenized in 20 mL sterile H_2_O using sterile quartz sand in individual mortars. The homogenates were centrifuged at 6000 × *g* for 10 min, and the supernatants were filtered through 0.22-μm membrane filters (Millipore, Billerica, MA, USA). The extraction sterility was tested by plating 100 μL of extract on nutrient agar and incubating at 37°C for 24 h. A 0.25-mL sample from each standardized bacterial culture (1 × 10^8^ cells mL^-1^) was used to inoculate 25 mL nutrient broth containing 200 mg L^-1^ *L*-tryptophan and 20% (v/v) sterile root extract. The cultures were incubated as described above and sampled every 6 h to measure melatonin production.

### Determination of MDA Content and H_2_O_2_ Accumulation

Fresh root samples were ground into a powder in liquid nitrogen, and H_2_O_2_ accumulation was quantified using a hydrogen peroxide assay kit (Beyotime, Shanghai, China), according to the manufacturer’s instructions. In brief, 0.1 g of root powder was homogenized in 1 mL phosphate buffer (50 mM; pH 6.0) at 4°C. The supernatant (50 μL) was mixed with 100 μL of test solution and placed at 30°C for 30 min. The absorbance was measured with a spectrometer at 560 nm and calibrated to a standard curve generated using known H_2_O_2_ concentrations.

In addition, 0.5 g of root powder was extracted with 2 mL of 10% (w/v) TCA to determine the MDA content of the grape roots as previously described (Zhao et al., 2013) with some modifications. After centrifugation at 8,000 × *g* for 10 min, 0.2 mL of the supernatant was added to the same volume of 0.5% (w/v) thiobarbituric acid in 20% (w/v) TCA, and was then heated at 100°C for 20 min. Reactions were stopped on ice. After centrifugation at 8,000 × *g* for 10 min, the absorbance was measured with a spectrometer at 440 nm, 532 nm, and 600 nm, and the MDA content (nmol g^-1^) was calculated as follows: [6.45 × (A532 – A600) – 0.56 × A450] × V/W, where V (mL) is the volume of the tissue extract, and W (g) is the FW.

### Quantitative Analysis of Melatonin and Its Intermediates Using UPLC-MS/MS

All standard reference materials were purchased from Sigma-Aldrich (St. Louis, MO, USA), and 15N double-labeled *L*-tryptophan was obtained from Cambridge Isotope Laboratories (Andover, MA, USA). The reagents, including methanol and formic acid (HPLC grade), were purchased from Merck (Darmstadt, Germany). Stock solutions were prepared by dissolving 10 mg of each standard in 1 mL of methanol under dim light conditions, and the stocks were then stored at -80°C to prevent degradation. Fresh working solutions were prepared in a 50:50 (v/v) solution of methanol:water.

Quantitative detection was conducted using a triple quadrupole UPLC-MS/MS (Agilent, Santa Clara, CA, USA). In the solvent system, eluent A was composed of Milli-Q water containing 0.1% (v/v) formic acid, and eluent B was methanol. Analyte separation was accomplished using an Agilent ZORBAX Eclipse XDB-C18 Rapid Resolution HT column (1.8 μm, 3.0 mm × 50 mm) at 42°C with linear elution gradient protocols of 0–6 min for 5–55% B and 6–15 min for 55–100% B at a flow rate of 0.2 mL min^-1^. Then, 100% B was kept constant for 2 min, and the column was re-equilibrated for 5 min using the initial solvent composition. The injection volume was 1 μL. Quantitation was determined using the multiple reactions monitoring mode under unit mass-resolution conditions (*L*-tryptophan *m/z*^+^ 205→188; ^15^N-*L*-tryptophan *m/z*^+^ 207→189; tryptamine *m/z*^+^ 161→144; ^15^N-tryptamine *m/z*^+^ 163→145; 5-hydroxytryptophan *m/z*^+^ 221→204; ^15^N-5-hydroxytryptophan *m/z*^+^ 223→205; serotonin *m/z*^+^ 177→160; ^15^N-serotonin *m/z*^+^ 179→161; *N*-acetylserotonin *m/z*^+^ 219→160; ^15^N-*N*-acetylserotonin *m/z*^+^ 220→161; melatonin *m/z*^+^ 233→174; and ^15^N-melatonin *m/z*^+^ 235→175).

### RNA Isolation and Quantitative Real-Time PCR Analysis

The expression levels of the grapevine tryptophan decarboxylase gene (*VvTDC*1) and the serotonin *N*-acetyltransferase gene (*VvSNAT*) in grape roots, previously predicted by Byeon et al. (2014a) and Jiao et al. (2016), were analyzed using quantitative real-time PCR (qRT-PCR). Root tissues were washed three times with sterile H_2_O, and RNA was extracted using the cetyl trimethylammonium bromide method (Reid et al., 2006). The RNA quantity was determined by measuring the A260 and A280 (NanoDrop^®^ ND-1000; Thermo Scientific, Wilmington, DE, USA). RNA was reverse transcribed into cDNA using the PrimeScript RT reagent kit with gDNA Eraser (TaKaRa, Dalian, China). qRT-PCR was conducted using a Roche 480 light cycler system with SYBR Fast qPCR Mix (TaKaRa) under the following conditions: 95°C for 30 s; followed by 40 cycles at 95°C for 5 s, 60°C for 10 s, and 72°C for 15 s. The melting curve analysis was performed at 95°C for 5 s, 60°C for 1 min, 95°C continuously, and then 50°C for 30 s. The primers used in this study are listed in **Table 1**. *EF1-*α was used as an internal reference to calculate the relative expression of each gene. All qRT-PCR reactions were performed in triplicate.

**Table 1:** Gene-specific primers used for quantitative real-time PCR.

Genes name	Description	GenBank no.	Forward primers (5′ to 3′)	Reverse primers (5’ to 3’)
*VvSNAT*	Serotonin *N*-acetyltransferase	XM_002266325	GCCCGTGCTACATCAGATCA	TTTGATGCCCTCTGGGTCAG
*VvTDC*1	Putative tryptophan decarboxylase-1	XM_010654123	CTGCCAGATTCCGCACCTAA	TCGCCGCAGGAGAAGTAATC
*EF-1*α	Elongation factor-1α	XM_002284888	GAACTGGGTGCTTGATAGGC	AACCAAAATATCCGGAGTAAAAGA

### Statistical Analysis

A correlation analysis based on a simple linear regression was performed on the assayed variables at the 95% confidence level between the relative expression of *VvTDC1* and *VvSNAT* and the MDA content. Data were expressed as the average of 3–12 replicates ± standard deviation. One-way analysis of variance (ANOVA) was performed in conjunction with Tukey’s test to detect melatonin intermediates in the E- roots of the four grape cultivars, and Student’s *t*-test was used to identify significant differences (*P* < 0.05) in bacterial colonization between treatments. All statistical analyses were performed using SPSS 19.0 (IBM, Armonk, NY, USA).

## Results

### Characterization of Melatonin Biosynthesis in *P. fluorescens* RG11

**FIGURE 2:**
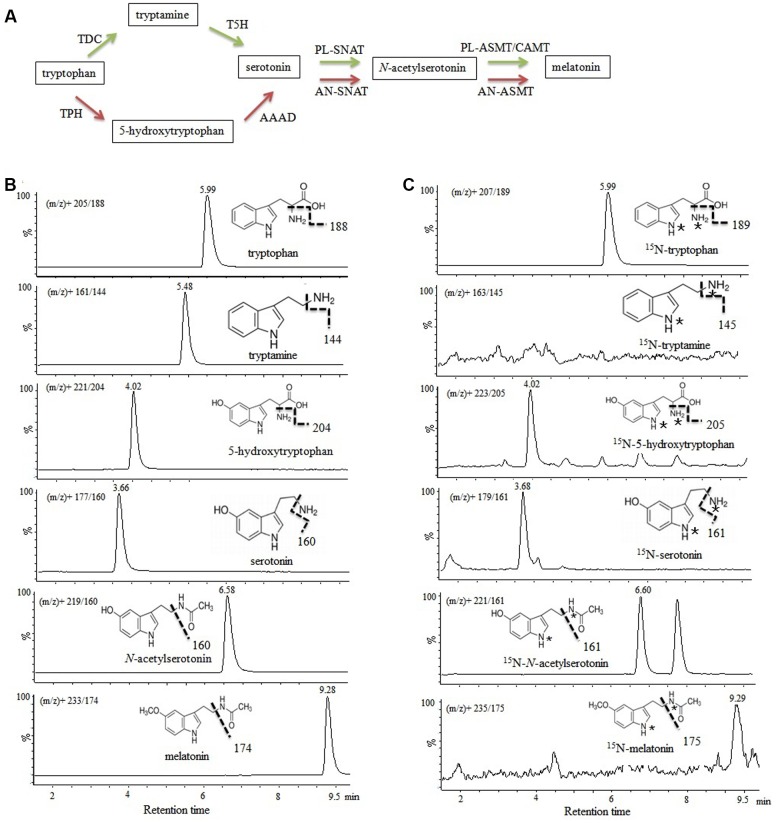
**Investigation of melatonin formation in *P. fluorescens* RG11 using 15N double-labeled *L*-tryptophan as the precursor**. **(A)** Biotransformation pathway of *L*-tryptophan to melatonin in animals (red) and plants (green). TPH, tryptophan hydroxylase; AAAD, aromatic amino acid decarboxylase; TDC, tryptophan decarboxylase; T5H, tryptamine 5-hydroxylase; AN-SNAT, animal serotonin *N*-acetyltransferase; PL-SNAT, plant serotonin *N*-acetyltransferase; AN-ASMT, animal *N*-acetylserotonin *O*-methyltransferase; PL-ASMT, plant *N*-acetylserotonin *O*-methyltransferase; CAMT, caffeic acid O-methyltransferase. **(B)** Chemical structures and chromatograms of six unlabeled standards (40–60 ng mL^-1^). **(C)** Chromatograms of ^15^N-tryptophan, ^15^N-5-hydroxytryptophan, ^15^N-serotonin, ^15^N-*N*-acetylserotonin, and ^15^N-melatonin in the *P. fluorescens* RG1 culture. ^15^N-tryptophan was converted to ^15^N-5-hydroxytryptophan, and ^15^N-tryptamine was not detected. ^∗^, ^15^N labeled.

The different melatonin synthetic pathways in animals and plants are presented in **Figure 2A**, and the chemical structures and chromatograms of six unlabeled standards (40–60 ng mL^-1^) and their corresponding ^15^N-metabolites are presented in **Figures 2B,C**. If tryptophan hydroxylase and tryptophan decarboxylase (or enzymes with similar activity) were present in *P. fluorescens* RG11, ^15^N-tryptophan could be converted to ^15^N-5-hydroxytryptophan (*m/z*^+^ 223→205) and ^15^N-tryptamine (*m/z*^+^ 163→145). A peak was obtained at *m/z*^+^ 223→205 with an identical retention time as that of the unlabeled 5-hydroxytryptophan standard, indicating that the compound was ^15^N-5-hydroxytryptophan. However, after RG11 was incubated with ^15^N-tryptophan, no peak was detected at the correct retention time for ^15^N-tryptamine at any time point of *m/z*^+^ 163→145 (**Figure 2C**). These findings suggest that 5-hydroxytryptophan might be a key intermediate in the melatonin biosynthesis pathway of *P. fluorescens* RG11. Similarly, the ^15^N-serotonin, ^15^N-*N*-acetylserotonin, and ^15^N-melatonin were all detected and confirmed using the reference compounds.

**FIGURE 3:**
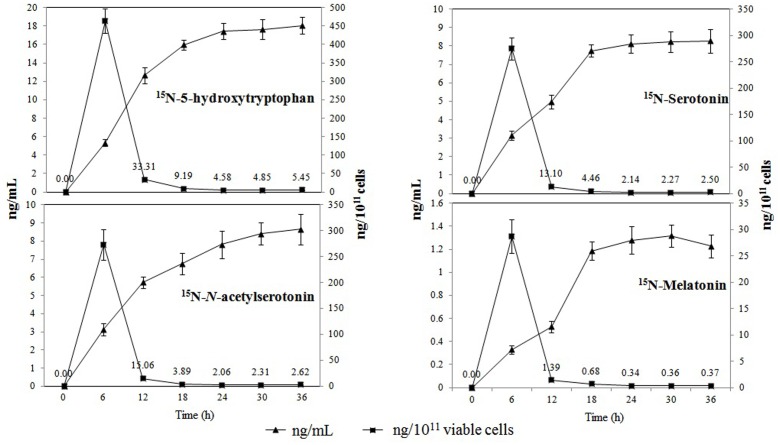
**Production of ^15^N-metabolites in the melatonin biosynthesis pathway of *P. fluorescens* RG11 in ng mL^-1^ (triangle) and ng 10^-11^ cells (square)**. Each data point represents the mean ± standard deviation (*n* = 3).

All of the ^15^N-metabolites were detectable 6 h post-incubation, and their levels showed a progressive increase over time (**Figure 3**). ^15^N-5-hydroxytryptophan, ^15^N-serotonin, and ^15^N-*N*-acetylserotonin were found at relatively higher concentrations that reached maximum values of 18.06 ± 1.14 ng mL^-1^, 8.28 ± 0.65 ng mL^-1^, and 8.66 ± 0.82 ng mL^-1^ at 36 h post-incubation, respectively. The concentrations of ^15^N-melatonin reached a maximum value of 1.32 ± 0.12 ng mL^-1^ at 30 h post-incubation and declined slightly thereafter. When the results were expressed in ng 10^-11^ viable cells, the production capacity for all metabolites peaked at 6 h post-incubation (cell number: 9.06 log 10 CFU mL^-1^) and subsequently declined with the increasing cell density (final cell number: 11.52 log 10 CFU mL^-1^).

### Changes in Endogenous Melatonin Levels and Its Intermediates in the Roots of E+ or E- Red Globe Plantlets under Salt Stress Conditions

**FIGURE 4:**
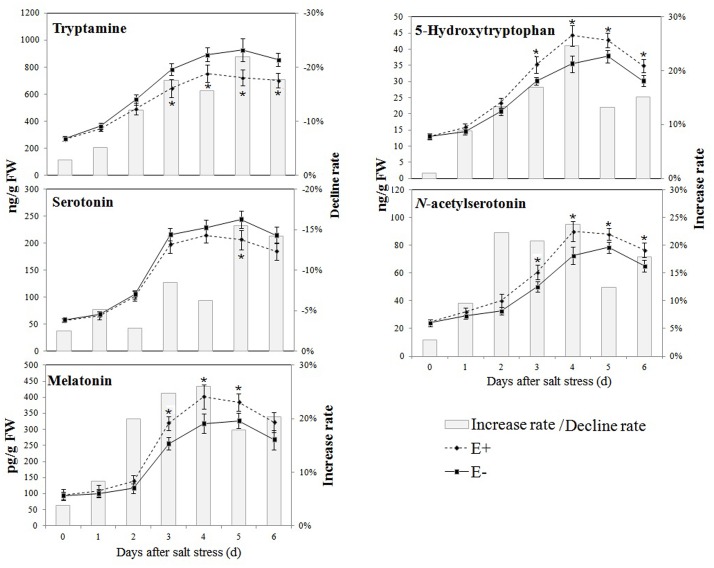
**Effect of *P. fluorescens* RG11 on the levels of endogenous melatonin and its intermediates in the roots of Red Globe plantlets under salt stress conditions**. E+, plants treated with RG11; E-, plants not treated with RG11. Changes in metabolite levels were calculated as follows: (E+/E-) – 1. Each data point represents the mean of 12 plants for each treatment ± standard deviation. Data with asterisks were significantly different from the E- plants at each time point (^∗^*P* < 0.05; Student’s *t*-test).

The effects of *P. fluorescens* RG11 on the levels of endogenous melatonin and its intermediates in the roots of Red Globe plantlets under salt stress are shown in **Figure 4**. The results show that the production of melatonin and its intermediates first increased in the roots of both E+ and E- plantlets and then decreased over time. The levels of 5-hydroxytryptophan, *N*-acetylserotonin, and melatonin in the roots of E+ plantlets were higher than those in the roots of E- plantlets, and the opposite trend was observed for levels of tryptamine and serotonin. The roots of E+ plantlets produced approximately 19.92–26.01% higher melatonin than those of E- plantlets between day 2 and day 6 of the salt stress treatment. The maximum value of melatonin in the roots of E+ plantlets (401.31 ± 37.78 pg g^-1^ FW) was found on day 4 of the salt stress treatment, whereas the maximum melatonin value of the roots of E- plantlets (326.66 ± 23.40 pg g^-1^ FW) was observed on day 5 of the salt stress treatment. Similar trends were also observed for 5-hydroxytryptophan and *N*-acetylserotonin, since their levels in the roots of E+ plantlets were 13.21–24.69% and 12.40–23.84% higher, respectively, compared with those in the roots of E- plantlets. These results indicate that the biosynthesis of melatonin in RG11-colonized plants responded earlier to salt stress, and the melatonin levels increased much more than E- plants. Conversely, the levels of tryptamine and serotonin in the roots of E+ plantlets were lower than those in the roots of E- plants, with a rate of decline of 12.10–21.98% and 6.23–15.54% between day 2 and day 6 of the salt stress treatment, respectively.

### Effect of *P. fluorescens* RG11 on Salt Stress-Induced Oxidative Damage and the Regulation of Melatonin-Related Genes in the Roots of E+ and E- Plantlets

**FIGURE 5:**
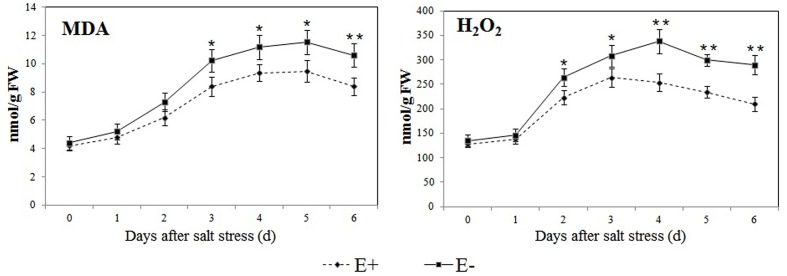
**Effect of *P. fluorescens* RG11 on salt-induced oxidative damage in the roots of E+ and E- ‘Red Globe’ plantlets**. Each data point represents the mean of 8 plants for each treatment ± standard deviation. Data with asterisks were significantly different from the E+ plants at each time point (^∗^*P* < 0.05; ^∗∗^*P* < 0.01; Student’s *t*-test).

The levels of MDA and H_2_O_2_ were similar in the roots of E+ and E- plantlets, but strongly increased after the salt stress treatment in all plantlets (**Figure 5**). However, E+ plantlets had levels that were 15.07–20.76% and 14.29–27.59% (*P* < 0.05) lower in MDA and H_2_O_2_, respectively, compared to E- plantlets between day 2 and day 6 of the salt stress treatment.

**FIGURE 6:**
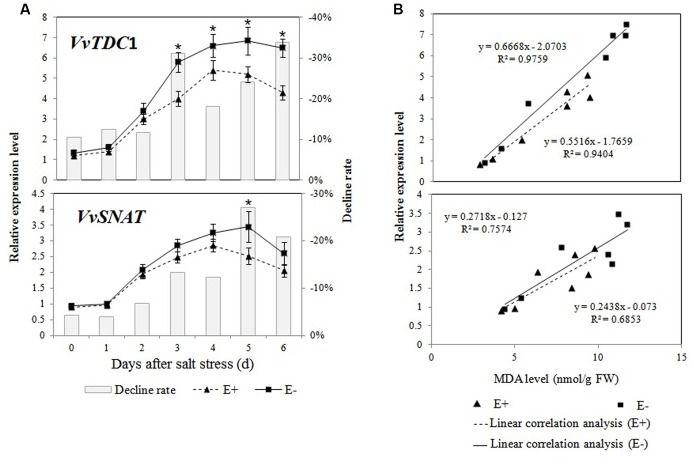
**Effects of *P. fluorescens* RG11 on the relative expression of *VvTDC*1 and *VvSNAT* in the roots of E+ and E- Red Globe plantlets under salt stress **(A)****. The results of a linear correlation analysis between the MDA content and the relative expression of *VvTDC*1 and *VvSNAT* **(B)**. Changes in metabolite levels were calculated as follows: (E+/E-) – 1. Each data point represents the mean of 12 plants in each treatment ± standard deviation. Data with asterisks are significantly different from the E+ plants at each time point (^∗^*P* < 0.05; Student’s *t*-test).

The expression profiles of *VvTDC*1 and *VvSNAT* in the roots of salt-treated plantlets are shown in **Figure 6A**. The qRT-PCR analysis demonstrated that the expression of *VvTDC*1 and *VvSNAT* first increased in the roots of all plantlets, and then decreased on day 5 or day 6 of the salt stress treatment. Changes in the expression of *VvTDC*1 and *VvSNAT* showed that the response of E+ plantlets to salt stress was slower and weaker compared to that of E- plantlets. However, the levels of endogenous melatonin in the roots of E+ plantlets were higher than those in the roots of E- plantlets.

Regression analyses showed a higher linear correlation coefficient between the relative expression of *VvTDC*1 and the MDA content (*R*^2^ = 0.9759 and 0.9404) in the roots of E+ and E- plantlets than that of *VvSNAT* (*R*^2^ = 0.7574 and 0.6853; **Figure 6B**). The results indicate that the melatonin synthesis genes, especially *VvTDC*1, might be induced by salt stress, and that the transcript levels were highly correlated with stress-induced oxidative damage.

### Effects of *P. fluorescens* RG11 Colonization on Plant Growth and Melatonin Biosynthesis in the Roots of Different Grape Cultivars under Salt Stress Conditions

Different grape hosts were used to assess the root colonization capacity of RG11, as well as its ability to promote plant growth. Root colonization of the inoculated endophyte is considered a prerequisite for successful growth promotion. We found that at 20 days post-inoculation, this strain successfully colonized the roots of all grape cultivars, and it was able to establish endophytic populations within the different cultivars with a density of approximately 5.44–6.05 Log 10 CFU g^-1^ FW.

**FIGURE 7:**
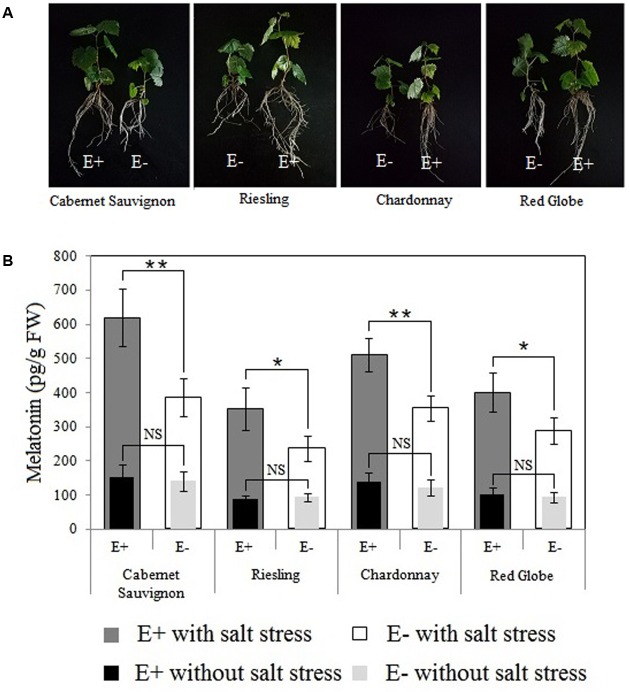
**Effects of *P. fluorescens* RG11 colonization on morphological differences between E+ and E- plantlets (A)** and the endogenous melatonin levels in the roots of the four grape cultivars under salt stress conditions **(B)**. E+, plants treated with the RG11; E-, plants not treated with the RG11. Each data point represents the mean of 12 plants for each treatment ± standard deviation. Data with asterisks indicate significant differences (^∗^*P* < 0.05; ^∗∗^*P* < 0.01; Student’s *t*-test). NS, not significant based on Student’s *t*-test.

Further, colonization with RG11 beneficially improved the growth of the grape plants as observed in the significant increase of growth attributes, although the extent of improvement varied between grape varieties (**Table 2**; **Figure 7A**). The increased ratios of root FW, total root length, and the number of lateral roots in relation to control plants ranged from approximately 25.98–38.85%, 18.51–44.41%, and 41.51–63.64%, respectively, and the highest ratio was found in the Red Globe cultivar. On the other hand, Cabernet Sauvignon displayed the highest increase in the ratio of plant height as well as in the chlorophyll content in leaves as compared to the other three varieties. Thus, it could be inferred that RG11 possesses a fairly broad plant growth promoting ability in different grape cultivars.

**Table 2:** Effects of *P. fluorescens* RG11 colonization on different growth attributes of Red Globe, Riesling, Chardonnay, and Cabernet Sauvignon grape cultivars 20 days post-inoculation (*n* = 12).

		Cabernet Sauvignon	Riesling	Chardonnay	Red Globe
Cell viability (Log 10 CFU/g FW)		5.44 ± 0.12	5.65 ± 0.25	5.76 ± 0.14	5.48 ± 0.18
Root fresh weight (g)	E+	3.26 ± 0.11^∗∗^	2.77 ± 0.10^∗^	2.88 ± 0.12^∗∗^	3.86 ± 0.15^∗∗^
	E-	2.54 ± 0.06	2.04 ± 0.08	2.26 ± 0.10	2.78 ± 0.14
	Increase ratio (%)	28.35	25.98	27.32	38.85^a^
Total root length (cm)	E+	345.40 ± 32^∗∗^	254.25 ± 22^∗^	325.64 ± 27^∗∗^	416.56 ± 36^∗∗^
	E-	258.25 ± 25	214.54 ± 18	258.53 ± 15	288.45 ± 24
	Increase ratio (%)	33.59	18.51	25.91	44.41^a^
Lateral root number	E+	12.4 ± 1.8^∗^	10.6 ± 0.74^∗∗^	15 ± 2.20^∗^	14.4 ± 1.5^∗∗^
	E-	8.1 ± 0.76	7.4 ± 0.65	10.6 ± 1.22	8.8 ± 0.66
	Increase ratio (%)	53.09	43.249	41.51	63.64^a^
Plant height (cm)	E+	12.06 ± 1.04^∗^	9.46 ± 0.82^∗^	8.48 ± 0.55^∗^	11.46 ± 0.96^∗^
	E-	9.44 ± 0.66	8.04 ± 0.60	7.46 ± 0.42	9.42 ± 0.54
	Increase ratio (%)	27.75^a^	17.67	13.67	21.66
Chlorophyll content (mg/g)	E+	1.55 ± 0.16^∗^	1.08 ± 0.08^∗^	1.14 ± 0.12	1.34 ± 0.15
	E-	1.29 ± 0.11	0.95 ± 0.10	1.02 ± 0.05	1.16 ± 0.13
	Increase ratio (%)	20.53^a^	14.16	11.76	15.52

*^a^The highest ratio of increase in growth attributes. E+, plants treated with RG11; E-, plants not treated with RG11. Data with asterisks were significantly different from the E- plants within each variety (^∗^*P* < 0.05; ^∗∗^*P* < 0.01; Student’s t-test)*.

The maximum difference in the levels of melatonin in E+ and E- Red Globe plantlets was detected at approximately day 4 after the start of the salt stress treatment (**Figure 4**), so the roots of E+ and E- plantlets were collected at this time for UPLC-MS/MS (**Figure 7B**). No significant differences were identified in the levels of melatonin between E+ and E- plantlets within each cultivar. However, all plants responded to salt stress by synthesizing melatonin, and *P. fluorescens* RG11 colonization increased the up-regulation of endogenous melatonin levels in the E+ plantlets of all cultivars as compared to E- plantlets. The levels of melatonin were higher (61.11%) in the roots of E+ Cabernet Sauvignon plantlets than in the roots of E- plantlets. Similar trends were observed in Red Globe, Riesling, and Chardonnay, although the increase was relatively lower. Therefore, *P. fluorescens* RG11 colonization might enhance the synthesis of melatonin in the roots of grape plantlets, especially in Cabernet Sauvignon, under salt stress conditions.

### Root Extraction Stimulated the Melatonin Biosynthesis of *P. fluorescens* RG11 *In vitro*

**FIGURE 8:**
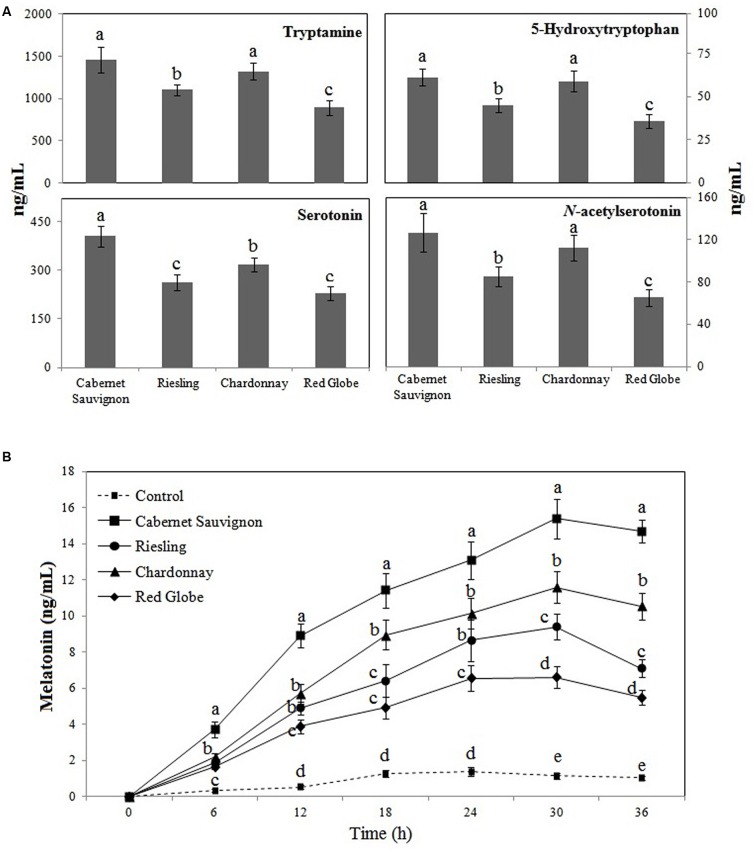
**The levels of tryptamine, 5-hydroxytryptophan, serotonin, and *N*-acetylserotonin in E- roots of Red Globe, Riesling, Chardonnay, and Cabernet Sauvignon grape cultivars under salt stress conditions (A)** and the effects of root extracts on *P. fluorescens* RG11 melatonin synthesis *in vitro* **(B)**. Each data point represents the mean ± standard deviation (*n* = 3). Different letters indicate significant difference based on Tukey’s multiple comparison test (*P* < 0.05); *n* = 3.

To further understand the relatively higher increase in melatonin production by RG11 colonization in Cabernet Sauvignon than in the other three varieties, we analyzed the levels of melatonin biosynthesis intermediates in the E- roots of the four grape cultivars (**Figure 8A**). At day 4 of the salt stress treatment, the levels of melatonin intermediates in the E- roots were different among the four grape cultivars: 894.55–1459.84 ng g^-1^ FW for tryptamine, 35.48–61.23 ng^-1^ FW for 5-hydroxytryptophan, 228.47–406.27 ng^-1^ FW for serotonin, and 82.87–126.65 ng^-1^ FW for *N*-acetylserotonin. Cabernet Sauvignon had the highest levels of tryptamine, 5-hydroxytryptophan, serotonin, and *N*-acetylserotonin, and these respective levels were 10.42–63.19%, 4.10–72.58%, 33.11–78.07%, and 15.42–52.83% higher than those in the roots of Chardonnay, Riesling, and Red Globe. These results suggest that genetic traits of the cultivars may noticeably influence the levels of melatonin intermediates in grapes.

The addition of root extracts collected from different cultivars significantly enhanced the melatonin synthesis of RG11 *in vitro* compared to the control (**Figure 8B**). The melatonin levels initially increased, but then slightly decreased in all cultures. The highest melatonin levels were detected after 24 h post-inoculation in control cultures and 30 h post-inoculation in cultures with root extracts. At 30 h post-inoculation, the levels of melatonin in cultures with root extracts from Red Globe, Riesling, Chardonnay, and Cabernet Sauvignon were higher by 5.75-fold, 10.11-fold, 8.19-fold, and 13.42-fold, respectively, as compared to the control (1.14 ± 0.16 ng mL^-1^). Noticeably, the root extracts of Cabernet Sauvignon had the strongest capacity to promote the melatonin synthesis of RG11 *in vitro*.

## Discussion

In a previous study, we characterized the potential melatonin synthesis in the endophytic bacterium *Bacillus amyloliquefaciens* SB-9 by detecting melatonin intermediates using *L*-tryptophan as a precursor (Jiao et al., 2016). Other melatonin intermediates, such as tryptamine and serotonin, were also found in small quantities in the untapped nutrient broth (data not reported), suggesting that beef extract or tryptone may possess these components. In the present study, we used 15N double-labeled *L*-tryptophan as the precursor of melatonin to investigate the biotransformation of ^15^N-tryptophan to ^15^N-melatonin in *P. fluorescens* RG11 in order to avoid the interference of intermediates that possibly existed in the nutrient broth. Additionally, this process allowed us to monitor the flow of isotopic tryptophan in the melatonin synthesis pathway. Although the genes involved in bacterial melatonin biosynthesis were not investigated in this study, to our knowledge, this is the first report on the flow of the carbon skeleton from tryptophan to melatonin in bacteria.

Isotopic tryptamine was not detected in the *P. fluorescens* RG11 culture, but the concentration of isotopic 5-hydroxytryptophan increased throughout the incubation period (**Figure 2**), indicating that the carbon skeleton of isotopic tryptophan was incorporated into 5-hydroxytryptophan rather than tryptamine by tryptophan hydroxylase (TPH, EC 1.14.16.4) or other enzymes with similar activity. However, the TPH genes, which catalyze the conversion of tryptophan into 5-hydroxytryptophan, were not found in the genome sequences of *P. fluorescens* deposited in GenBank. TPH belongs to the class of pterin-dependent aromatic amino acid hydroxylases (AAAHs) that include other two subgroups: phenylalanine hydroxylases (PAH, phenylalanine 4-monooxygenase, EC 1.14.16.1) and tyrosine hydroxylases (TH, EC 1.14.16.2) (Kappock and Caradonna, 1996). We found AAAHs are widely distributed in bacteria, but so far, the most of them have been identified as PAH. Some bacterial PAH homolog genes in *Chromobacterium violaceum* (Kino et al., 2009) and *P. aeruginosa* (Zhao and Jensen, 1994) have been reported to catalyze the conversion of tryptophan into 5-hydroxytryptophan. We, therefore, speculated that PAH might be responsible for the formation of 5-hydroxytryptophan, which is the first step of melatonin biosynthesis in *P. fluorescens* RG11. In several *Pseudomonas* species, including *P. fluorescens*, PAH has been confirmed to catalyze tryptophan hydroxylation (Lin et al., 2014). Further studies using knockout mutants of the PAH gene are needed to determine the correlation between PAH and bacterial melatonin production.

Our data also show that the concentration of isotopic melatonin and its intermediates progressively increased between 0 and 30 h post-incubation; however, the production capacity peaked at 6 h and then sharply declined in the growth phase of *P. fluorescens* RG11 (**Figure 3**). These results are consistent with our previous report on *B. amyloliquefaciens* SB-9 (Jiao et al., 2016). The studies of melatonin in other bacteria are currently limited, but melatonin in yeast was found to be mainly produced during the exponential growth phase (Rodriguez-Naranjo et al., 2012; Vigentini et al., 2015; Fernández-Cruz et al., 2016). Tan et al. (2015) hypothesized that melatonin primarily functions as a free radical scavenger and antioxidant in unicellular organisms, and other functions were acquired during the evolution of multi-cellular organisms. Consistent with what has been demonstrated in yeast, our results also indicate that melatonin could serve as a bacterial growth-signaling molecule or as protection against ROS in the medium to facilitate early adaptability.

Previous studies showed *P*. *fluorescens* strains induced systemic resistance in plants (Ramamoorthy et al., 2001; Wang et al., 2005; Saravanakumar and Samiyappan, 2007) and decreased oxidative damage by enhancing plant antioxidant enzyme activities and/or increasing the production of phenolic compounds or other antioxidants (Torres et al., 2012). The results of the present study concur with those of previous reports, and we found that after exposure to salt stress, oxidative damage in the roots of E+ Red Globe plantlets was decreased by RG11 colonization (**Figure 5**). Thus, it would be expected that the levels of melatonin and its intermediates in the roots of E+ plantlets would be lower than those in the roots of E- plantlets, because the transcript levels of melatonin synthesis genes, *VvTDC*1 and *VvSNAT*, were relatively lower than those in E- plantlets (**Figure 6A**). The upregulation of these genes is positively correlated with ROS levels caused by abiotic stress (Li et al., 2014). However, we observed that only tryptamine and serotonin followed this trend, whereas the levels of melatonin and other intermediates were higher in the roots of E+ plantlets (**Figures 4** and **7**). The higher endogenous 5-hydroxytryptophan, *N*-acetylserotonin, and melatonin levels in the roots of these plantlets resulted from RG11 colonization, which might compensate for the production of these compounds via a supplemental bacterial melatonin biosynthesis pathway, a possible exchange of metabolites between the plant and endophyte, or additional promoting factors produced by the endophyte. In fact, in the present study, RG11 was able to secrete all of these compounds *in vivo*, with the exception of tryptamine. Nevertheless, we still have no direct evidence that the enhanced levels of endogenous melatonin were derived from production by endophytic bacteria.

Previous studies also showed that *P*. *fluorescens* promotes plant growth by affecting various traits, including nitrogen fixation, phosphorus solubilization, production of 1-aminocyclopropane-1-carboxylate deaminase, or induction of physical and chemical (gibberellins and auxin) changes (Jaleel et al., 2007; Saravanakumar and Samiyappan, 2007; Etesami et al., 2014). We found that *P*. *fluorescens* RG11 exhibited a fairly broad plant growth-promoting ability (**Table 2**; **Figure 7A**). Although we cannot be sure whether *P*. *fluorescens* RG11 simultaneously possesses the traits listed above, we believe that the growth-promoting ability observed in different grapes may be derived from the bacteria’s combined effects. Moreover, the increased melatonin levels from RG11 colonization in E+ roots might partly contribute to growth promotion, since melatonin has been reported to stimulate plant growth in several plants, even at low concentrations (Chen et al., 2009; Park and Back, 2012; Bajwa et al., 2014; Wei et al., 2014; Arnao and Hernández-Ruiz, 2016).

Additionally, *P*. *fluorescens* RG11 enhanced the production of endogenous melatonin in the roots of different grape cultivars under salt stress conditions, especially that of Cabernet Sauvignon (**Figure 7B**). The root tissue fluid is the natural source of nutrients for endophytes that produce phytohormones that induce a physiological response in the host plant (Kamilova et al., 2006). Therefore, RG11 might utilize melatonin precursors produced by root tissues to expedite its own melatonin biosynthesis inside the roots, and the intraspecific variation of these metabolite levels or other promoters between the roots of the four grape cultivars could be responsible for the relatively high differences in the melatonin levels of E+ plants. The roots of Cabernet Sauvignon exhibited the highest concentrations of melatonin precursors, and its root crude extracts significantly induced RG11 melatonin biosynthesis *in vitro* compared to the control and other grape cultivars (**Figure 8**).

## Conclusion

The results of this study revealed a potential melatonin synthesis pathway in bacteria and functions for the enhancement of endogenous melatonin in plants, which are crucial for understanding plant–rhizobacteria interactions and improving the application of melatonin-producing endophytes in agriculture. The successful utilization of melatonin-synthesizing bacteria in agriculture requires a thorough understanding of the mechanisms that increase the levels of endogenous melatonin in plants. However, further studies are needed to verify the functions of melatonin-related genes in endophytic bacteria.

## Author Contributions

CL, YM, and JJ conceived the study; YM and JJ performed the experiments, analyzed the data, and wrote the manuscript; JJ analyzed the UPLC-MS/MS data; XF, YZ, JJ, and HS provided suggestions and revised the manuscript. All authors approved the final manuscript and agreed to be accountable for all aspects of the work, thus ensuring that questions related to the accuracy or integrity of any part of the work are appropriately investigated and resolved.

## Conflict of Interest Statement

The authors declare that the research was conducted in the absence of any commercial or financial relationships that could be construed as a potential conflict of interest.

## Abbreviations

H_2_O_2_: hydrogen peroxide
MDA: malondialdehyde
MS: Murashige and Skoog
ROS: reactive oxygen species
TCA: trichloroacetic acid
UPLC-MS/MS: ultra-performance liquid chromatography-tandem mass spectrometry.
